# Sensitive Dentine and Its Remedy

**Published:** 1874-04

**Authors:** E. M. Morrison

**Affiliations:** Des Moines, Iowa


### SENSITIVE DENTINE AND ITS REMEDY.

BY E. M. MORRISON, M. D., DES MOINES, IOWA.

In the DENTAL REGISTER for February, 1874, we find an article under the above caption, and as the author criticises freely, it is presumed that he will not object to being criticised himself.

The most vital part of the subject is clouded and vacant, and we are left to grope our way in darkness where " we do most need the light." We have been told, that to be successful in the administration of medicines, it was essential to know what to give, how to give, and when to quit giving; and, as remedies are given, or should be given, or applied for the effects they produce, in cases of this kind, where, there is danger of destroying important vital organs, the most essential point is to know when to quit, but here the light fails to fall upon our dim pathway, and the vagueness of the long past is only intensified.

The author says of the arsenic: " Remove it as soon as the effect is accomplished." But how are we to know when the effect is accomplished ? We are not informed, but here is the nearest approach in the article, to the solution of the question: " If a hard and dense tooth, four hours sufficed, if a delicate, frail, translucent tooth, two hours. If the first application did not prove sufficient, apply again, but for a less time." This does not satisfy our wants however, but he further says: "I should remark here that the first effect of the arsenic is to increase the sensitiveness, but by letting the tooth remain open for two or three days, this entirely disappears." Are we to estimate the sufficiency of the action of the remedy by the intensity of the "increased sensitivness?" Where is the line of demarcation, and what are the symptoms pointing to the precise time when we shall remove the arsenic, and not endanger the pulp of the tooth ?

Prof. Tomes' caution on this subject, to say the most of it, is not more dangerous than the gentleman's befogged shoals in the approach to his happy haven, in which he has successfully anchored his *barque* for the last ten years. We do not like the engineering, however, that carries the captain safely into port, and leaves the crew among the breakers, in darkness and uncertainty, without chart or compass.

If we cut with a surgeon's knife, we can see where the blade separates the tissues; the line that divides the living from the dead is distinct and well defined; but the means of knowing how far we destroy the "Sentient ends of the fibrille," with arsenic, and when to remove it, and not endanger the pulp of the tooth, is not so apparent. We want certainty, no guessing on two or four hours, or repetitions, to indicate to us when we shall lift the dark mantle of death that we place over the pulps of the teeth entrusted to our care for treatment and preservation. Time tells all things, but in death, and what would destroy the pulp of one tooth, or the life of one individual in a few minutes, might be borne for hours by others, or with impunity by some. Effects and the means of knowing them with certainty, and not the number of hours, should guide our action. Our knowledge should be correct, and our skill directed as unerringly as Wm. Tell's arrow, then we would be able to apply arsenic, if the premises be true, with tolerable certainty, and see the *death line* under our control; the skull and crossbones might be laid aside, and we could say in the semblance of truth at least: "Thus far shalt thou go and no further."

